# Experience and Attitudes towards Information Technology among First-Year Medical Students in Denmark: Longitudinal Questionnaire Survey

**DOI:** 10.2196/jmir.6.1.e10

**Published:** 2004-03-05

**Authors:** Jens Dørup

**Affiliations:** ^1^Section for Health InformaticsUniversity of AarhusDenmark

**Keywords:** Information technology, Internet, e-mail, students, medical, education, medical

## Abstract

**Background:**

As more and more information technology (IT) resources become available both for support of campus- based medical education and for Web-based learning, it becomes increasingly interesting to map the information technology resources available to medical students and the attitudes students have towards their use.

**Objective:**

To determine how extensively and effectively information handling skills are being taught in the medical curriculum, the study investigated Internet and computer availability and usage, and attitudes towards information technology among first-year medical students in Aarhus, Denmark, during a five-year period.

**Methods:**

In the period from 1998 to 2002, students beginning the first semester of medical school were given courses on effective use of IT in their studies. As a part of the tutorials, the students were asked to complete a web-based questionnaire which included questions related to IT readiness and attitudes towards using IT in studies.

**Results:**

A total of 1159 students (78%) responded. Overall, 71.7% of the respondents indicating they had access to a computer at home, a number that did not change significantly during the study period. Over time, the power of students' computers and the use of e-mail and Internet did increase significantly. By fall 2002, approximately 90% of students used e-mail regularly, 80% used the Internet regularly, and 60% had access to the Internet from home. Significantly more males than females had access to a computer at home, and males had a more positive attitude towards the use of computers in their medical studies. A fairly constant number of students (3-7%) stated that they would prefer not to have to use computers in their studies.

**Conclusions:**

Taken together with our experience from classroom teaching, these results indicate optional teaching of basic information technology still needs to be integrated into medical studies, and that this need does not seem likely to disappear in the near future.

## Introduction

Software for computer-assisted learning (CAL) in medicine has been available from the 1960s, or almost as long as computers. Results of evaluations of software for teaching and learning in medicine vary considerably, largely because of differences in the learning situations and in the evaluation design [1-5]. In spite of the early awareness of the potential of CAL programs among dedicated educators, production and marketing of a substantial amount of programs started only after the multimedia computer became readily available in the mid-1990s.

With the increased availability and speed of the Internet come new ways of disseminating interactive multimedia learning modules and possibilities for integrating information and communication technologies into other methods of teaching and learning.

The approach of many teachers and course directors in traditional universities has been to "wait and see." A growing number of enthusiasts, however, have tried to implement new ways of teaching and learning using, or facilitated by, information technology (IT), and in many cases integration of information and communication technology (ICT) has been used to support changes in teaching and learning methods.

The approach of students, whose motivation is usually exams, could also be characterized as "wait and see." The question is how long to wait and then what to look for. Access to computers, at home and on campus, and Internet access are both important. The issue of students' IT literacy, however, may be even more important. It is often thought that it is only a matter of time until all young students become skilful in the use of computers, and IT training is no longer necessary in higher education. The present study was conducted to test this hypothesis and to determine whether students entering medical school already had all the IT skills they would need to use IT effectively during their medical studies. Analysing IT skills is especially difficult when comparing results over a span of years: what would be seen as a trivial task today may have required advanced knowledge five years ago. Consequently, instead of skills, the study investigated computer availability and attitudes and how these measures might be interrelated. Several previous studies have addressed the issue of IT literacy among medical students, typically through questionnaires given in one or a few semesters [6- 14]. The present study, extending from the 1998 fall semester to the 2002 fall semester, revealed developments over a five-year period that was characterized by an unforeseen expansion in IT and Internet availability throughout the Western world.

We asked medical students about their access to IT, their attitudes and interests in using IT as part of the medical studies they had just started. The purpose was to clarify when and where IT could be effectively integrated and what IT training and hardware needs could be expected.

## Methods

In the period from 1998 to 2002 (nine semesters), students beginning the first semester of medical school in Aarhus, Denmark, were given two lectures and three group-based tutorials on effective use of IT in their studies. As a part of the tutorials, the students were asked to complete a questionnaire (seAppendix 1). This Web-based questionnaire was completed 2 to 3 weeks after start of studies, and included questions related to IT readiness and attitudes towards using IT in studies. The questionnaire was set up using Coldfusion [15] on a Windows 2000 www-server. Data were entered into an open database connectivity (ODBC) database and were analysed using Microsoft Excel. Teachers were present during the input phase to answer technical questions.

The students were asked their age and sex; whether they had access to a computer at home and whether it was PC, Macintosh, or other; which operating system it used; the amount of RAM; the processor type; and whether the computer had a soundcard, a CD drive, or a modem attached. Students were also asked whether they had access to the Internet and e-mail from home, and the frequency of usage. Finally, the students were asked the following questions about their attitudes towards IT in the medical school.

1. Would you like to use the computer for calculations and reports?
2. Would you like to use the computer as a supplement to teaching?
3. Would you like to use the computer as a replacement for some of the theoretical teaching?
4. Would you like to be able to ask questions to teachers via e-mail?
5. Would you like to be able to use the computer for distance learning from home?
6. Would you prefer that you *did not* have to use the computer during your medical studies?

Minor additions to answering options were made as new processors and operating systems became available.

The Χ ^2^ test was used to compare frequencies.

## Results

### Basic data

**Figure 1a-f:**
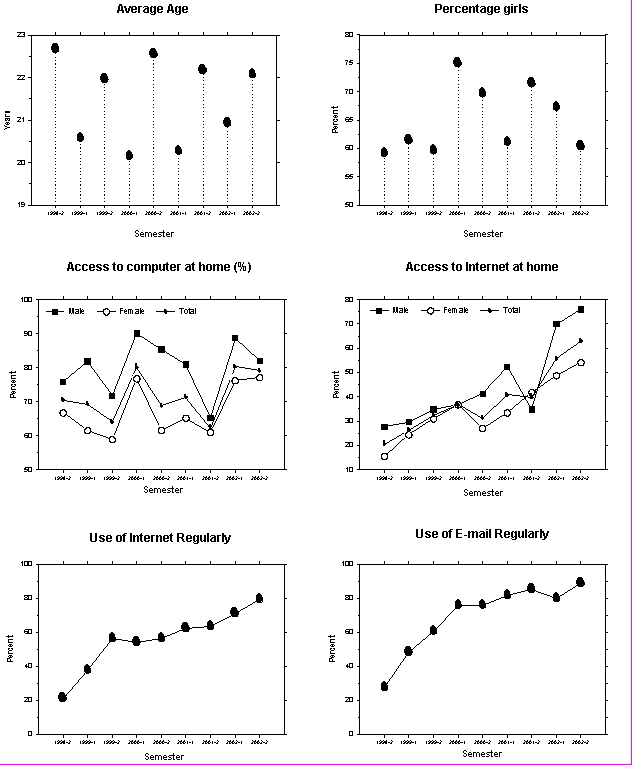
Distribution of age and gender (a, b) and computer, Internet, and e-mail usage (c to f). Spring semesters are indicated with *1*; fall semesters with *2*. Use of Internet and e-mail was graded as never, rarely, regularly, often, or daily. The curves sum up answers: regularly, often, and daily

Of 1474 medical students who started their studies between fall 1998 and fall 2002, 1159 (78.6%) completed the Web-based questionnaire. The average age was 21.6 years (Figure 1b). Students beginning in the summer were, on average, 2 years older than those beginning in winter, which is a result of the way students are accepted for medical studies in Aarhus. Of all participating students, 60.3% were female. Distributions for semesters are given in Figure 1a and Figure 1b. A total of 71.7% of all students (79.7% of males; 67.5% of females) indicated they had access to a computer at home. There were substantial fluctuations from semester to semester (Figure 1c), but comparing the average of the first four semesters with the average of the last four revealed only a slight increase from 70.9% to 73.1% (NS). The increase from 70.4% in fall 1998 to 79.3% in 2002 was not statistically significant. Internet access from home increased from 20.4% to 62.9% (p < 0.001) in the study period, and there was an even more pronounced increase in the use, from any location, of Internet and e-mail (Figure 1e and Figure 1f). It is especially significant that, although the university did not require the students to use e-mail, by the end of the study period, 88.7% of all students used e-mail regularly.

### Computers used

**Figure 2a-f:**
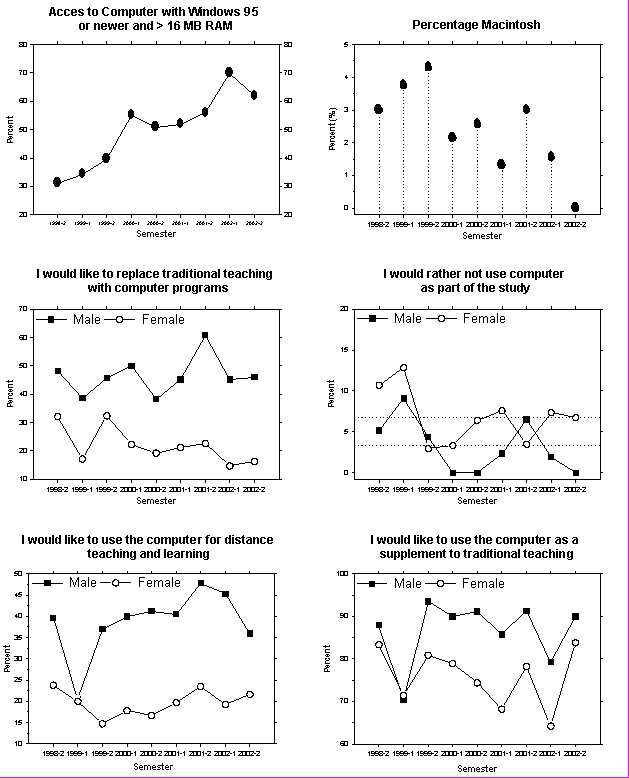
Type of computer (a, b) and attitudes towards use of computer (c to f). Spring semesters are indicated with *1*; fall semesters with *2*. As might be expected, the number of powerful computers among students was increasing; The percentage of Macintosh computers was decreasing. 6.8% of females answered that they would rather not use a computer as part of the study. The corresponding percentage for males was 3.3%

Not surprisingly, the power of computers increased in the study period (Figure 2a). The incidence of soundcards in computers available to students increased from 48% to 71% (p < 0.001), the incidence of CD drives in home computers increased from 52% to 76% (p < 0.001), and of modems from 27% to 59% (p < 0.001).

Among medical students in Denmark, Macintosh computers are not in common use. Numbers decreased from about 3 to 4% at the start of the study period to about 0 to 1% in the last part (Figure 2b).

### Attitudes

Males were significantly (p < 0.001) more interested in replacing traditional with ICT- supported teaching and learning. An average of 46.6% of males versus 21.8% of females indicated that they would like to replace traditional teaching with use of computers if possible. Despite fluctuations, there was no trend towards a consistent change in these numbers during the study period (Figure 2c).

A small percentage (6.8% of females and 3.3% of males) indicated that they would prefer not to have to use a computer during their medical studies. Again, in spite of fluctuations, there was no trend towards a consistent change in this number (Figure 2d).

The difference between males and females in attitudes towards use of distance education was also highly significant (p < 0.001): 38.7% of males versus 19.9% of females indicated a positive attitude (Figure 2e).

The attitude towards use of ICT resources as a supplement was more positive than for ICT replacement or for distance education (86% of boys and 76% of girls were positive, Figure 2f)

## Discussion

Use of IT as a tool to support medical teaching and learning was introduced gradually over several years at The University of Aarhus, without a well-defined strategy. Similar trends have been reported from many other universities. One reason may be the great diversity of IT skills among both teachers and students. There may also be a general reluctance to change educational methods and a belief that available IT resources would not enhance learning. Many things changed during the period of this study. Many medical schools have moved towards problem-based learning. Implementation of computer-supported collaborative learning [16] has driven IT investments and implementation in some schools, and certainly the value of available Internet resources and of e-communication in supporting medical learning can no longer be denied. At the University of Aarhus, which is a typical research-intensive institution, educational methods have undergone an evolution rather that a revolution and IT has not been considered a driving force for pedagogical change. Still, project work and other study areas that rely heavily on medical literature available through the Web have demonstrated the need for a minimum of IT literacy among the students, and it is important to know roughly the number of students in need of special IT courses.

This study shows that an average of 71.7% of new medical students had access to a computer at home. The number was significantly higher among males, and showed considerable fluctuations from semester to semester, but did not increase significantly in the study period (between 1998 and 2002). When compared to German study from 1992 by Gunther Eysenbach, however, this study shows a significant increase in IT availability and knowledge [17].

Approximately 79% of registered students completed the questionnaire. The seminar included a number of important practical issues, such as a general introduction to the use of the university's IT facilities. We encouraged all participants in the seminars to complete the survey, but did not record reasons for not completing it. In principle, the 21% of registered students who did not complete the questionnaire might have different attitudes towards IT and their access to computers at home might differ from those who completed the survey. If this was the case, absolute numbers might be slightly biased. We do, however, assume that failure to complete the survey would be fairly constant during the study period, and thus relative developments with time would be unaffected by the missing questionnaires.

As noted, students beginning medical studies in the summer were an average of 2 years older than those beginning in the winter. A comparison of computer availability with age, revealed a U-shaped curve: ~80% at 19 years, ~60% at 22 years, and ~88% at 26 years. Changes in computer availability during the study period therefore can probably be attributed, at least in part, to fluctuations in age. Fluctuations in the percentage of females accepted could also be expected to influence computer availability over years. A comparison of Figure 1b and Figure 1c, however, does not show a consistent trend.

The most comprehensive study of IT literacy conducted in European universities is probably the Survey of European Universities Skills in ICT of Students and Staff (SEUSISS) project, funded under the EU-Socrates Minerva Programme. The consortium, which includes seven universities from the UK, Finland, Norway, The Netherlands, Italy, France, and Spain, gathered data in 2001 and 2002 in a continuation of a 10-year University of Edinburgh data collection project [18,19,20]. Although the present study included only medical students, many of the questions related to IT literacy can be expected to apply to all students in higher education, with the exception of students in computer science and related areas.

Questions that remain important in the present study and in the SEUSISS and Edinburgh studies are:

- What are the developments in *general IT literacy*, computer availability at home, and general availability to IT, and can we expect that problems with IT literacy will disappear with time?
- Can university teachers and administrators expect that *e-communication* will reach all students?
- Are *gender differences* in IT attitudes and literacy significant and do these differences tend to change over time?

### General IT literacy

Roughly half of all students had access to a home-PC with a CD, soundcard, and sufficient computing power and RAM to effectively run modern multimedia applications. Whereas access to *any* PC at home showed only a very small increase, access to a "modern multimedia PC" increased from about 30% to about 65%. In the SEUSISS project (2001-2002), the numbers for PC ownership at the start of studies varied from 54% (Åbo, Finland) to 89% (Groningen, the Netherlands). In Edinburgh by 2000, ownership was at 44% for males and 37% for females, and the Edinburgh study reports that this gender gap diminished with time. The data from this study seem to be consistent with the other European data, although the gender differences in this study did not decrease, and no distinction was made in this study between ownership and availability of IT at home. Availability of a powerful PC at home is increasing, but a substantial proportion of new students still do not have access to a computer at home, and it is still too early to predict that they will within the next few years.

The Edinburgh study reports about 8% Macintosh users, and a slight trend towards more Macintosh users among women. Medical students, like most other computer users in Denmark, use PCs almost exclusively.

It is one thing is to own a PC or to have access to one, quite another is to possess IT skills and to be IT literate. A dramatic increase in IT literacy might be expected during these years when IT and Internet use have increased generally. Data on Internet availability are continuously monitored by the European Union, and their data show that Denmark is one of the three leading countries with respect to IT use and Internet spread [21].

In the present study, direct measures of IT-literacy were not identified, and no attempt was made to test the students in this respect. However, it seems reasonable to expect a correlation between attitudes towards IT and IT literacy. Indeed in hands-on courses a small percentage of students were found to be absolute novices with respect to computer use. Between 3 and 7% of the students (significantly more females than males) who indicated that they would prefer not to have to use computers in their studies. The consistency of this finding, corroborated in several earlier studies [7,8,9,14,18], suggests there is a need for training in basic IT and information-handling skills as an optional element in medical training [22]. Moreover, this study does not suggest this need is likely to disappear in the near future.

### E-communication

Use of Internet and e-mail increased dramatically during the study period. The curve for e-mail, in approaching 100%, shows a decreasing slope, while the curve for Internet use indicates that a further increase above 80% can be expected. An interesting question is whether these numbers will increase to 100%. At present, the university does not use e-mail and Internet as a mandatory route of communication with or among medical students, but the numbers presented here indicate that the time may be ripe to start doing so. Several universities around the world have successfully started using e-mail as a mandatory communication, and the Internet as a mandatory information and communication channel. Although a small number of students and teachers may experience going from paper-based to e-communication as a drastic step, this study shows that it will probably not be a problem for the vast majority, and that the minority will meet only temporary difficulties.

### Gender differences

This study has revealed significant gender differences both in access to computers and in readiness to integrate IT in the learning process. As an example, roughly 50% of males versus 25% of females responded that they would like to replace some traditional teaching with IT-based activities.

In these respects, the study correlates directly with the 10 year follow-up study from Edinburgh [18,19]. The results are interesting in a number of ways when it comes to planning, developing, and implementing ICT-supported learning activities. Communications with the medical students indicate that most female students are not directly opposed to ICT-supported teaching and learning; however, female students may be more pragmatic and more focused on exams, whereas some male students may favour the freedom of time and space offered by e-learning.

### Conclusions

During the five years of this study there was a dramatic increase in availability of IT resources. It can be considered positive when about 30% of students indicated that they would like to exchange traditional learning for e-learning, and about 80% indicated that would like to use ICT resources as a supplement. Some course directors and teachers are still reluctant to increase the importance of IT support in the studies, but this study indicates that most students are ready to change. There is still a need for teaching in basic IT as part of medical study, and this need does not seem likely to disappear in the near future.

## Multimedia Appendix

Web-based Questionnaire:

## Abbreviations

CAL: computer assisted learning
EU: European Union
IT: information technology
ICT: information and communication technology
ODBC: open database connectivity
RAM: random access memory
SEUSISS: Survey of European Universities Skills in ICT of Students and Staff
