# Supplementary material for: Experience and Attitudes towards Information Technology among First-Year Medical Students in Denmark: Longitudinal Questionnaire Survey
Source: J Med Internet Res. 2004 Mar 5;6(1):e10. doi: 10.2196/jmir.6.1.e10 (PMC1550582; doi:10.2196/jmir.6.1.e10)
Supplement: Supplementary file 1 [file jmir_v6i1e10_app1.html]

| Age |  | Years |  |  |  |  |  |  |
| Sex | Female | Male |  |  |  |  |  |  |
| Have you got access to a computer at home ? |  |  |  |  |  |  |  |  |
| Which type of computer | PC | Mac | Other | None |  |  |  |  |
| Operating System | DOS | Win3.x | 95/98/Me | NT/2000/XP | MacOS | Other | None |  |
| Processor \*Including corresponding Cyrix or AMD | 286-386\* | 486\* | Pentium\* | PII\* | PIII/IV\* | Other | None |  |
| How much RAM memory? | ââ¤ 1MB | 1-4 | 5-16 | 17-31 | 32-64 | 65-256 | > 256 | None |
| Does your PC have a soundcard? |  |  |  |  |  |  |  |  |
| Does your PC have a CD ROM drive? |  |  |  |  |  |  |  |  |
| Do you have a modem connected (incl: ISDN, ADSL, Cable Modem or LAN)? |  |  |  |  |  |  |  |  |
| Have you got Internet access at home? |  |  |  |  |  |  |  |  |
| Use of Internet from home? | Never | Rarely | Regularly | Often | Daily |  |  |  |
| Have you got an e-mail address |  |  |  |  |  |  |  |  |
| Use of Email? | Never | Rarely | Regularly | Often | Daily |  |  |  |
| I like to use a computer for calculations and reports |  |  |  |  |  |  |  |  |
| I wished I *would not have* to use a computer as part of my medical studies |  |  |  |  |  |  |  |  |
| I would like to use the computer as a supplement to other teaching activities |  |  |  |  |  |  |  |  |
| I would like to use the computer in stead of other teaching activities |  |  |  |  |  |  |  |  |
| I would like to use e-mail to ask questions to my teachers if possible |  |  |  |  |  |  |  |  |
| I would like to use the computer for distance education from home |  |  |  |  |  |  |  |  |

  
Comments 
